# Inhibiting the redox function of APE1 suppresses cervical cancer metastasis via disengagement of ZEB1 from E-cadherin in EMT

**DOI:** 10.1186/s13046-021-02006-5

**Published:** 2021-07-01

**Authors:** Qing Li, Zhi-Wei Zhou, Wei Duan, Cheng-Yuan Qian, Shu-Nan Wang, Meng-Sheng Deng, Dan Zi, Jian-Min Wang, Cheng-Yi Mao, Guanbin Song, Dong Wang, Kenneth D. Westover, Cheng-Xiong Xu

**Affiliations:** 1 Cancer Center, Daping Hospital, Army Medical University, Chongqing, 400042 China; 2 School of Medicine, Chongqing University, Chongqing, 400030 China; 3 State Key Laboratory of Trauma, Burn and Combined Injury, Research Institute of Surgery, Army Medical University, Chongqing, 400042 China; 4 Department of Radiation Oncology and Biochemistry, University of Texas Southwestern Medical Center, Dallas, TX 75390 USA; 5 Jingzhou Central Hospital, The Second Clinical Medical College, Yangtze University, JingZhou, 434020 Hubei China; 6 Department of Radiology, Daping Hospital, Army Medical University, Chongqing, 400042 China; 7 Department of Obstetrics and Gynecology, Affiliated Hospital of Guizhou Medical University, Guiyang, 550004 Guizhou China; 8 Department of Pathology, Daping Hospital, Army Medical University, Chongqing, 400042 China; 9 Key Laboratory of Biorheological Science and Technology, Ministry of Education, College of Bioengineering, Chongqing University, Chongqing, 400030 China

**Keywords:** APE1, EMT, E-cadherin, ZEB1, Cervical cancer metastasis

## Abstract

**Background:**

Metastasis is a major challenge in cervical cancer treatment. Previous studies have shown that the dual functional protein apurinic/apyrimidinic endonuclease 1 (APE1) promotes tumor metastasis and is overexpressed in cervical cancer. However, the biological role and mechanism of APE1 in cervical cancer metastasis have rarely been studied.

**Methods:**

We used gene set enrichment analysis (GSEA) to determine the APE1-related signaling pathways in cervical cancer. To investigate the role and mechanism of APE1 in cervical cancer metastasis and invasion, immunohistochemistry, immunofluorescence, western blotting, secondary structure prediction, coimmunoprecipitation, luciferase reporter, and electrophoretic mobility shift assays were performed. The inhibitory effects of the APE1 redox function inhibitor APX3330 on cervical cancer metastasis were evaluated using animal models.

**Results:**

Clinical data showed that high expression of APE1 was associated with lymph node metastasis in cervical cancer patients. GSEA results showed that APE1 was associated with epithelial to mesenchymal transition (EMT) in cervical cancer. Ectopic expression of APE1 promoted EMT and invasion of cervical cancer cells, whereas inhibition of APE1 suppressed EMT and invasion of cervical cancer cells in a redox function-dependent manner. Notably, APE1 redox function inhibitor APX3330 treatment dramatically suppressed cervical cancer cell lymph node and distant metastasis in vivo. Furthermore, we found that APE1 enhanced the interaction between ZEB1 and the E-cadherin promoter by binding to ZEB1, thereby suppressing the expression of E-cadherin, a negative regulator of EMT.

**Conclusion:**

Our findings help to elucidate the role played by APE1 in cervical cancer metastasis and targeting APE1 redox function may be a novel strategy for inhibiting cervical cancer metastasis.

**Supplementary Information:**

The online version contains supplementary material available at 10.1186/s13046-021-02006-5.

## Background

Cervical cancer is the third most common cancer and the second leading cause of cancer-related death in women worldwide [1, 2]. The major challenge in cervical cancer treatment is metastasis because most of the mortality associated with cervical cancer is caused by metastasis [3]. Unfortunately, no effective therapeutic strategies exist for preventing or inhibiting cervical cancer metastasis, in part because the mechanisms that underlie metastasis are incompletely understood.

Growing evidence illustrates that epithelial-mesenchymal transition (EMT) plays a key role in tumor metastasis [4–7]. EMT is a process in which epithelial tumor cells lose their cell polarity and cell-cell adhesion and gain migratory and invasive properties [8]. Notably, the clinical data show that EMT is closely related to a poor prognosis in cervical cancer patients [9, 10]. E-cadherin is a homotypic cell-to-cell adhesion molecule ubiquitously expressed on epithelial cells [11]. However, E-cadherin is frequently downregulated in cervical cancer [12], and downregulation of E-cadherin is sufficient to induce EMT in tumor cells and promote tumor cell metastasis [13, 14]. Importantly, clinical data show that low expression of E-cadherin is closely associated with metastasis and a poor prognosis in cervical cancer patients [15, 16].

In cancer, one cause of downregulation of E-cadherin is abnormal overexpression of zinc finger E-box-binding homeobox 1 (ZEB1). ZEB1 is a transcriptional repressor that inhibits E-cadherin expression at the transcriptional level by binding to the E-cadherin promoter [17]. According to Chen et al., ZEB1 is not expressed in normal cervical epithelial cells but is expressed in most invasive cervical carcinomas, and the ZEB1 expression level is strongly associated with lymph node metastasis in cervical cancer patients [7]. Consistent with this, ZEB1 silencing inhibits cervical cancer cell EMT and metastasis [18], suggesting that ZEB1 is a potential therapeutic target for EMT-induced cervical cancer metastasis treatment. Unfortunately, no specific ZEB1 inhibitors are available.

Apurinic/apyrimidinic endonuclease/redox factor-1 (APE1/Ref-1) functions both as a redox regulator of transcription factor activation and as part of the DNA damage response [19]. Notably, recent studies showed that upregulated APE1 inhibits E-cadherin expression and stimulates EMT and metastasis in non-small-cell lung cancer [20, 21]. In addition, Wei et al. reported that upregulated APE1 was closely associated with lymph node metastasis in gastric cancer [22]. These findings suggest that APE1 may be involved in the regulation of E-cadherin-mediated EMT and tumor metastasis, but the mechanism is unclear. Aberrantly upregulated expression of APE1 has also been detected in cervical cancer [23], but the effects of APE1 on cervical cancer metastasis have not been studied.

In this study, we found that high expression of APE1 was closely associated with EMT and lymph node metastasis in cervical cancer patients. Ectopic expression of APE1 inhibited E-cadherin expression and stimulated EMT and invasion in cervical cancer cells. In contrast, inhibition of APE1 redox function significantly suppressed lymph node and distant metastasis of cervical cancer cells in vivo. Furthermore, we found that APE1 inhibited E-cadherin expression in a redox-dependent manner by enhancing the interaction between ZEB1 and the E-cadherin promoter by directly binding to ZEB1. In summary, our findings provide new insights into the underlying mechanism of metastasis in cervical cancer and provide a potential therapeutic target for metastatic cervical cancer therapy.

## Materials and methods

### Cell culture and human specimens

HeLa and SiHa cells were obtained from the American Type Culture Collection (ATCC, Manassas, VA, USA) and maintained in Dulbecco’s modified Eagle’s medium with 10% fetal bovine serum (HyClone, Logan, UT). Human samples were obtained from patients with cervical cancer by biopsy or surgery at Daping Hospital and Research Institute of Surgery. This study was approved by the Ethics Committee of Daping Hospital, Army Medical University. We obtained consent to publish from the participants (or legal parents or guardians for children) to report individual patient data.

### Plasmid constructs

The ZEB1 ORF was amplified by PCR from human cDNAs and inserted into the *BamH*I and *Hind*III sites of the pcDNA3.1-flag vector for expression in mammalian cells. The E-cadherin promoter region from − 670 to + 92 was amplified using primers with restriction enzyme sites *Bgl*II or *Hind*III at each end and inserted into the upstream region of the firefly luciferase gene of the pGL3-Basic vector (Promega, Madison, WI, USA) [24]. APE1 (NM_001641.4) and ZEB1 (NM_001323642.2) expression vectors were obtained from Shanghai GeneChem Co., Ltd. (Shanghai, China). All primer sequences used in this study are given in Table S1.

### Immunohistochemistry (IHC), immunofluorescence (IF), and western blot

For western blotting, cells were lysed in RIPA lysis buffer (Thermo Fisher Scientific, Waltham, MA, USA) supplemented with protease and phosphatase inhibitor cocktails (Sigma-Aldrich, Saint Louis, MO, USA), and the protein concentration of the lysate was measured using a Bradford kit (Bio-Rad, Hercules, CA, USA). Equal amounts of proteins (30 μg) were separated by sodium dodecyl sulfate–polyacrylamide gel electrophoresis and transferred to nitrocellulose membranes. The membrane was blocked for 1 h in Tris-buffered saline with Tween 20 (TBST) containing 5% skim milk at room temperature (RT), and immunoblotting was performed by incubating the membranes overnight with their corresponding primary antibodies in 5% skim milk at 4 °C. The membrane was washed with TBST and then incubated with horseradish peroxidase (HRP)-conjugated secondary antibodies for 1 h at RT. After washing, the proteins were visualized with an enhanced chemiluminescence detection kit (Thermo Fisher Scientific) in accordance with the manufacturer’s recommendations.

For IHC, the tumor tissues were fixed in 10% buffered formalin (Sigma-Aldrich), embedded in paraffin, and sectioned at 4 μM. The tissue sections were deparaffinized in xylene, rehydrated through an alcohol gradient, washed and incubated in 0.3% hydrogen peroxide (AppliChem, Darmstadt, Germany) for 15 min. After washing, the tissue sections were blocked with 5% bovine serum albumin in PBS for 1 h. Then, primary antibodies were applied to the tissue sections overnight at 4 °C. The following day, the tissue sections were washed and incubated with secondary HRP-conjugated antibodies for 1 h at RT and counterstained with Mayer’s hematoxylin (Dako, Carpinteria, CA, USA) for 10 s. Coverslips were mounted using Permount (Thermo Fisher Scientific). A panel of pathologists reviewed the IHC staining and scored it as follows: score 0, no staining positive tumor cells; score 1, staining positive tumor cells less than 10% of the total tumor cells; score 2, staining positive tumor cells more than 10% of the total tumor cells, but less than 50%; and score 3, staining positive tumor cells more than 50% of the total tumor cells. Scores of 0 and 1 were defined as low expression, and scores of 2 and 3 were defined as high expression.

For IF, cells were grown on coverslips and transfected with the indicated oligonucleotides. After 72 h of transfection, the cells were fixed with 4% paraformaldehyde for 15 min and then permeabilized with 0.3% Triton X-100 for 10 min. The cells were washed with PBS and incubated with 3.5% bovine serum albumin (BSA) for 1 h followed by incubation with the primary antibody in 3.5% BSA for 1 h at RT. The cells were washed with PBS and incubated with FITC-conjugated secondary antibody for 1 h at RT in the dark. Then, the cells were counterstained with 4,6-diamidino-2-phenylindole (DAPI) (Sigma-Aldrich) for 30 min. Antibodies against APE1, β-actin, E-cadherin, N-cadherin, vimentin, Flag, HA, and GAPDH and secondary antibodies were purchased from Abcam (Cambridge, MA, USA). The anti-ZEB1 antibody was obtained from Cell Signaling Technology (Danvers, MA, USA).

### Invasion assay

Cells were transfected with APE1 construct or siRNA (GeneChem Co., Shanghai, China) using Lipofectamine 3000 (Invitrogen, Carlsbad, CA, USA) according to the manufacturer’s instructions. Sequences of the double-stranded siRNAs are antisense (5′-GUCUGGUACGACUGGAGUACC-3′, 5′-UACUCCAGUCGUACCAGACCU-3′) and nonsense (5′-CCAUGAGGUCAGCAUGGUCUG3’, 5′-GACCAUGCUGACCUCAUGGAA-3′) [21]. Nontargeting control siRNA was purchased from Qiagen (Hilden, Germany). After 48 h of transfection, the cells were subjected to invasion assays. Briefly, 10,000 cells in medium without serum were seeded in the upper wells of invasion chambers (BD Biosciences, San Jose, CA, USA). The lower wells contained the same medium supplemented with 10% fetal bovine serum. After 24 h, the cells that invaded to the other side of the chamber were fixed with 2.5% glutaraldehyde, stained with 0.1% crystal violet, and counted.

To investigate the effects of the APE1 redox inhibitor APX3330 (Selleck, Houston, TX, USA) and the APE1 DNA repair inhibitor APE1 inhibitor III (Merck Millipore, Molsheim, France) on the invasion of cervical cancer cells, cells were pretreated with APE1 inhibitors for 24 h. After 24 h, the cells were subjected to invasion assays as described above.

### Luciferase reporter assay

Plasmids and/or nucleotides were transfected into HeLa cells that were transfected with a firefly luciferase reporter construct containing the E-cadherin promoter. The Renilla luciferase plasmid was cotransfected as a transfection control (Promega). The cell extracts were processed 72 h after transfection, and the luciferase activity was measured using the Dual-Luciferase Reporter Assay System (Promega) according to the manufacturer’s instructions. The luciferase activity was normalized to the activity of Renilla luciferase.

### Co-immunoprecipitation

HeLa cells were transfected with the indicated plasmids. After 72 h, the cells were lysed in lysis buffer [1% Nonidet P40, 0.1% SDS, 50 mM Tris-HCl (pH 7.8), 150 mM NaCl, 1 mM DTT and 0.5 mM EDTA containing protease inhibitors], and the protein concentration of the lysate was measured using a Bradford kit (Bio-Rad). Immunoprecipitation was performed using monoclonal antibodies as indicated. Immunocomplexes were collected on protein A/G-agarose beads (Merck Millipore), washed twice with lysis buffer, and then washed twice with wash buffer [10 mM Tris (pH 7.4), 1 mM EDTA, 1 mM EGTA (pH 8.0), 150 mM NaCl, 1% Triton X-100, 0.2 mM sodium orthovanadate, protease inhibitor cocktail]. The beads were dissolved in SDS loading buffer (Bio-Rad), denatured at 95 °C for 10 min, and later subjected to western blot analysis.

### Secondary structure prediction

The sequence of ZEB1 was retrieved from UniProt, and the corresponding secondary structure was generated using Jpred (Jnet version: 2.3.1) (http://www.compbio.dundee.ac.uk/jpred/).

### Electrophoretic mobility shift assay (EMSA)

EMSA was performed using the LightShift chemiluminescence EMSA kit (Thermo Fisher Scientific) according to the manufacturer’s instructions as described previously [25]. Nuclear extracts were isolated from the indicated cells and incubated with 3′-biotin-labeled double-stranded oligonucleotide probes containing consensus sequences for ZEB1 binding sites (Fig. S1), then the samples were separated on a 5% polyacrylamide gel and transferred to a Zeta-Probe GT nylon membrane. The probes were detected by HRP-conjugated streptavidin. The probe sequences are shown in Table S1.

### Animal experiments

All xenograft models were generated using GFP-expressing HeLa cells (HeLa-GFP) in 6-week-old female BALB/c nude mice. For the lymph node metastatic model, HeLa-GFP cells (1 × 10^6^/50 μl PBS per mouse) were directly injected into the footpad of the mice [26]. For the abdominal cavity metastatic model, HeLa-GFP cells (1 × 10^6^/200 μl PBS per mouse) were intraperitoneally (IP) injected into the mice. For the lung metastatic model, HeLa-GFP cells (1 × 10^6^/100 μl PBS per mouse) were injected intravenously into the tail vein of the mice. One week after the cell injection, the mice were randomly divided into two groups. The control group mice were treated with PBS, and the treatment group mice were treated with APX3330 (12.5 mg/kg body weight) by IP injection once every 2 days. The mice were treated with APX3330 for 3 weeks for the lung and lymph node metastasis experiments, treated with APX3330 for 2 weeks in the abdominal cavity metastasis experiments, and their body weight was measured every 3 days. Tumor metastasis was monitored by an IVIS Spectrum In Vivo Imaging System (Perkin Elmer, Utah, USA) and MRI (7.0-T MRI, Bruker Biospec 70/20USR, Germany). All animal experiments complied with the Daping Hospital, Army Medical University Policy on the Care and Use of Laboratory Animals.

### Statistical analyses

All statistical analyses were performed using Prism 5.0 software (GraphPad). The unpaired two-samples t-test was used to compare the mean of two independent groups, and one-way ANOVA was used to determine differences between the means of two or more independent groups. *p* values less than 0.05 were considered statistically significant. The data are presented as the mean ± the standard deviation (SD) of at least three independent experiments. The correlation between APE1 expression and lymph node metastasis or E-cadherin expression was tested by the chi-square test.

## Results

### High expression of APE1 is closely associated with EMT and lymph node metastasis in cervical cancer patients

**Fig. 1:**
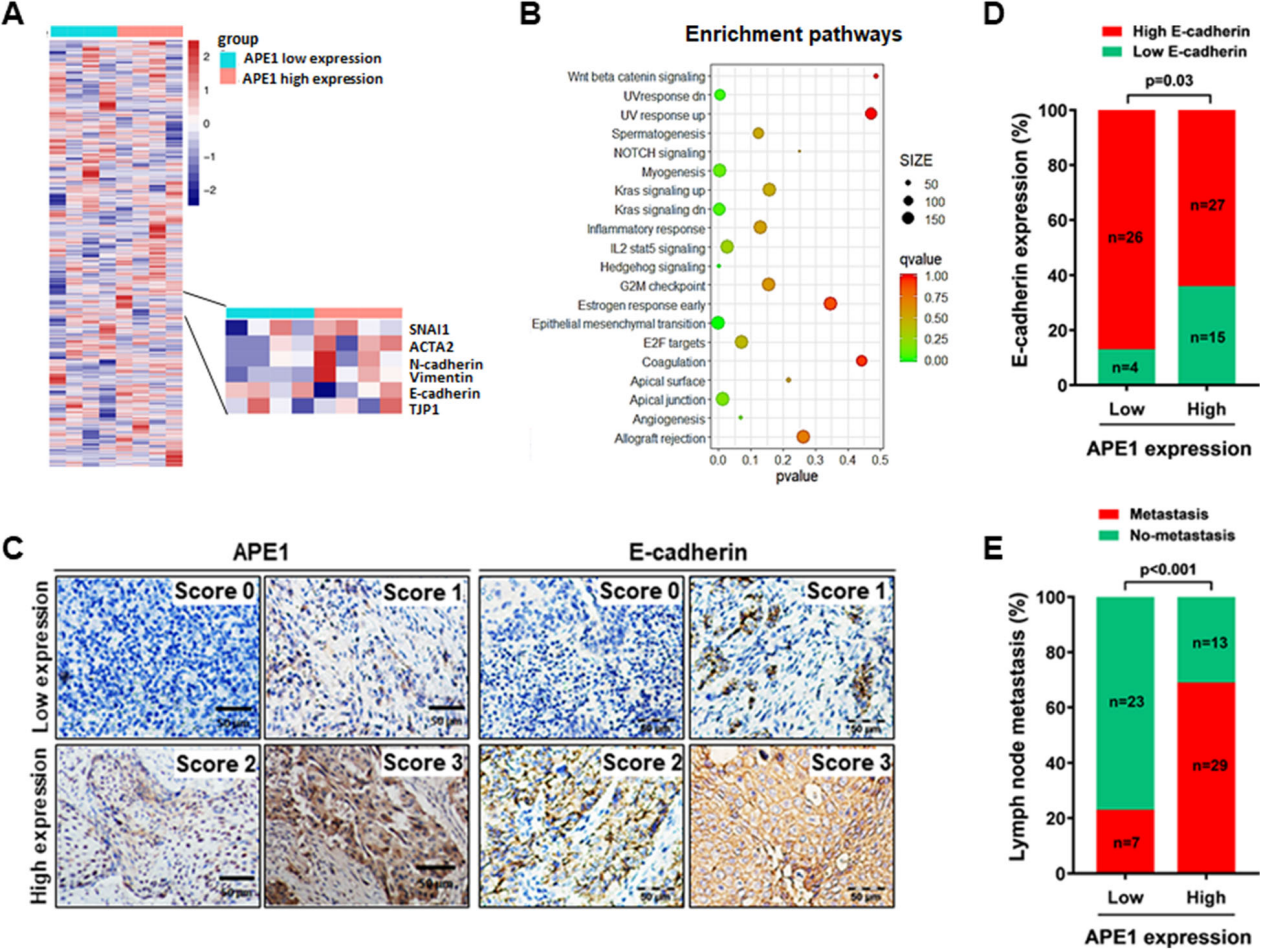
APE1 expression associated with EMT and lymph node metastasis in cervical cancer patients. **a** Heatmap showing genes differentially expressed in cervical cancer with high expression (*n* = 4) and low expression of APE1 (*n* = 4). If the APE1 expression level in cancer tissues was higher than adjacent tissues, it was classified as the APE1 high expression group and if there was no difference, it was classified as the APE1 low expression group. **b** Gene set enrichment analysis (GSEA) of signaling pathways. GSEA was performed using transcriptome data from cervical cancer samples with high expression (*n* = 4) and low expression of APE1 (*n* = 4). If the mRNA expression level of APE1 in cancer tissues was higher than adjacent tissues, it was classified as the APE1 high expression group and if there was no difference, it was classified as the APE1 low expression group. **c** The expression levels of APE1 and E-cadherin were determined by immunohistochemistry (IHC) of 72 specimens of cervical cancer patients. The representative images are the standard scoring images used to evaluate the intensity of APE1 and E-cadherin staining. **d** The expression of APE1 and E-cadherin was negatively associated in cervical cancer patients. **e** A high expression level of APE1 was correlated with lymph node metastasis in cervical cancer patients. The difference was tested by the chi square test

To investigate whether the high expression of APE1 in cervical cancer is related to EMT and metastasis, we performed gene set enrichment analysis (GSEA) using transcriptome data from four cervical cancer samples expressing high levels of APE1 and four cervical cancer samples expressing low levels of APE1 (Fig. 1a and Fig. S2). The GSEA results showed that the APE1 expression level was closely associated with EMT in cervical cancer (Fig. 1b). This association was further confirmed in an expanded 72 cervical cancer patient cohort where we directly compared APE1 to E-cadherin (loss of E-cadherin is a well-established hallmark of EMT) expression using IHC (Fig. 1c). Our data showed that among the 30 cases with low APE1 expression, 26 cases (87%) showed high expression of E-cadherin, while among the 42 cases with high APE1 expression, 27 (64%) cases presented with high expression of E-cadherin (Fig. 1d). This result suggests that high expression of APE1 is associated with EMT in cervical cancer patients.

Because EMT stimulates cancer metastasis partially through the lymphatic system [27, 28], we next evaluated the associations between APE1 expression and lymph node metastasis. The results showed that 69% (29 cases) of cases with high APE1 expression (42 cases) had lymph node metastasis, while only 23% (7 cases) of cases with low APE1 expression (30 cases) had lymph node metastasis (Fig. 1e and Table 1). Taken together, these findings indicate that APE1 may be involved in metastasis by stimulating EMT in cervical cancer patients.

**Table 1:** Characteristics of patients with cervical cancer

Variable	Number of patients (%)		*p* value
	APE1 high	APE1 low	
Age(years)			0.54
≥ 50	14 (33%)	8 (27%)	
<50	28 (67%)	22 (73%)	
Histological subtypes			0.56
Squamous carcinoma	34 (81%)	27 (90%)	
Adenocarcinoma	6 (14%)	2 (7%)	
Adenosquamous	2 (5%)	1 (3%)	
Tumor grade			0.11
Poorly differentiation	28 (67%)	14 (48%)	
Moderate differentiation	10 (24%)	8 (26%)	
High differentiation	4 (9%)	8 (26%)	
Stage			0.94
Stage I	23 (55%)	16 (53%)	
Stage II	14 (33%)	11 (37%)	
Stage III	5 (12%)	3 (10%)	
Lymph node metastasis			< 0.01
Positive	29 (69%)	7 (23%)	
Negative	13 (31%)	23 (77%)	

### Ectopic expression of APE1 stimulates EMT and the invasion of cervical cancer cells

**Fig. 2:**
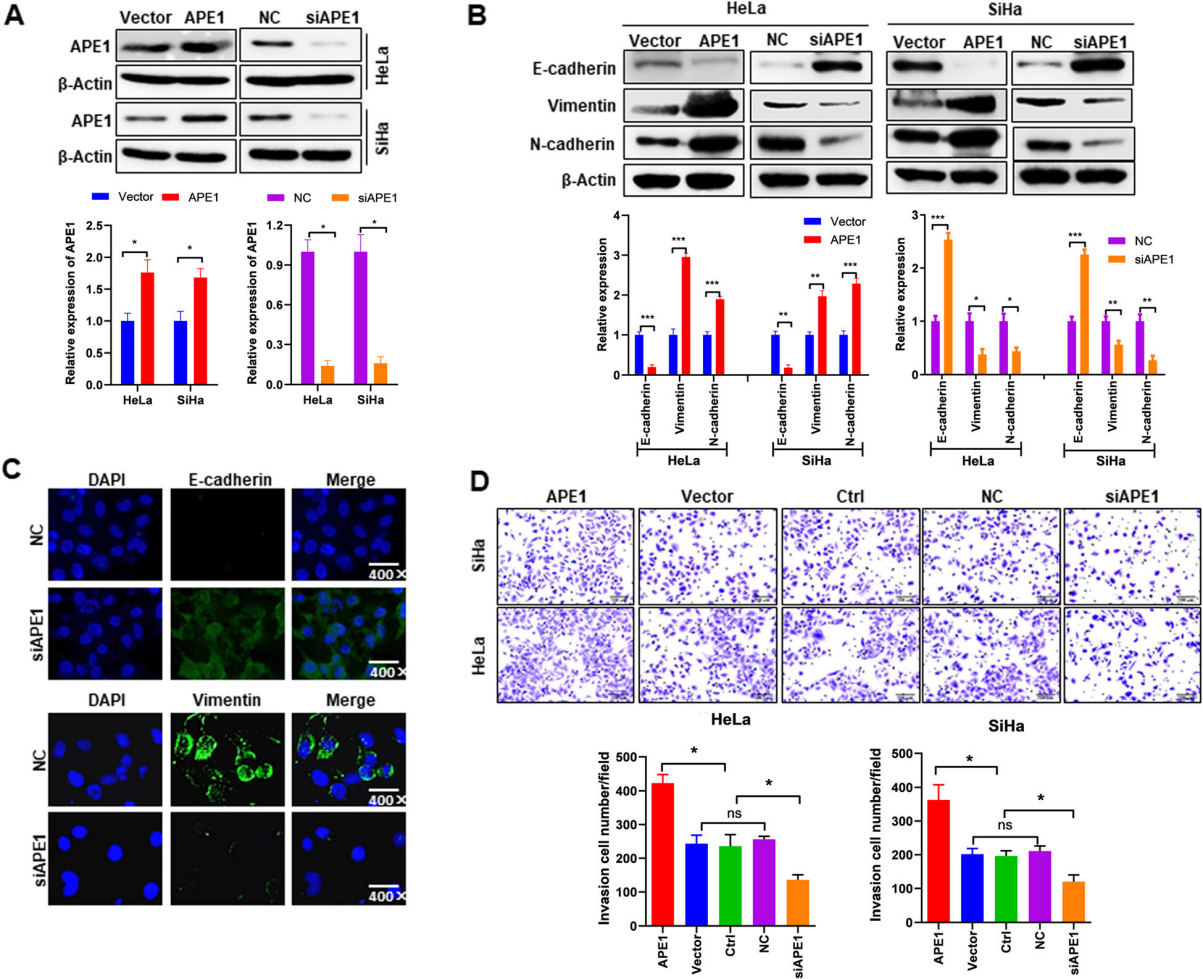
APE1 stimulates EMT and invasion of cervical cancer cells. **a** APE1 expression levels detected by western blot. Indicated cells were transfected with the indicated plasmid or siRNA. Then, 72 h after the transfection, the cells were subjected to western blot analysis. **b** Representative blots showing that APE1 positively regulates EMT in cervical cancer cells. Western blotting was performed 72 h post-transfection. **c** Immunofluorescence (IF) showing that silencing APE1 stimulates E-cadherin expression and inhibits vimentin expression in HeLa cells. HeLa cells were transfected with the indicated siRNAs. After 72 h of transfection, IF analysis was performed. **d** Cervical cancer cell invasion was stimulated or inhibited by the overexpression or knockdown of APE1, respectively. Vector, cells transfected with empty vector; APE1, cells transfected with APE1 expression plasmid; NC, cells transfected with nontargeting control siRNA; siAPE1, cells transfected with APE1 siRNA; Ctrl, cells not treated with anything. *, *P* < 0.05; **, *P* < 0.01; ***, *P* < 0.001

To investigate whether APE1 directly stimulates EMT in cervical cancer cells, leading to metastasis, both HeLa and SiHa cells were transfected with APE1-expressing plasmid or APE1 siRNA (Fig. 2a) and then subjected to detection of EMT-related protein expression and invasion assays. As shown in Fig. 2b, western blot analysis showed that ectopic expression of APE1 inhibited the epithelial cell marker E-cadherin but upregulated the mesenchymal marker proteins vimentin and N-cadherin in both HeLa and SiHa cells. In contrast, silencing APE1 upregulated E-cadherin but downregulated vimentin and N-cadherin expression (Fig. 2b). Consistent with this, IF also showed that silencing APE1 stimulated E-cadherin expression but inhibited vimentin expression in HeLa cells (Fig. 2c). Of functional importance, cervical cancer cell invasion was significantly increased by ectopic expression of APE1 but suppressed by silencing of APE1 (Fig. 2d). Taken together, these findings indicate that APE1 positively regulates cervical cancer cell EMT and invasion.

### APE1 promotes cervical cancer cell EMT and invasion via a redox-dependent mechanism

**Fig. 3:**
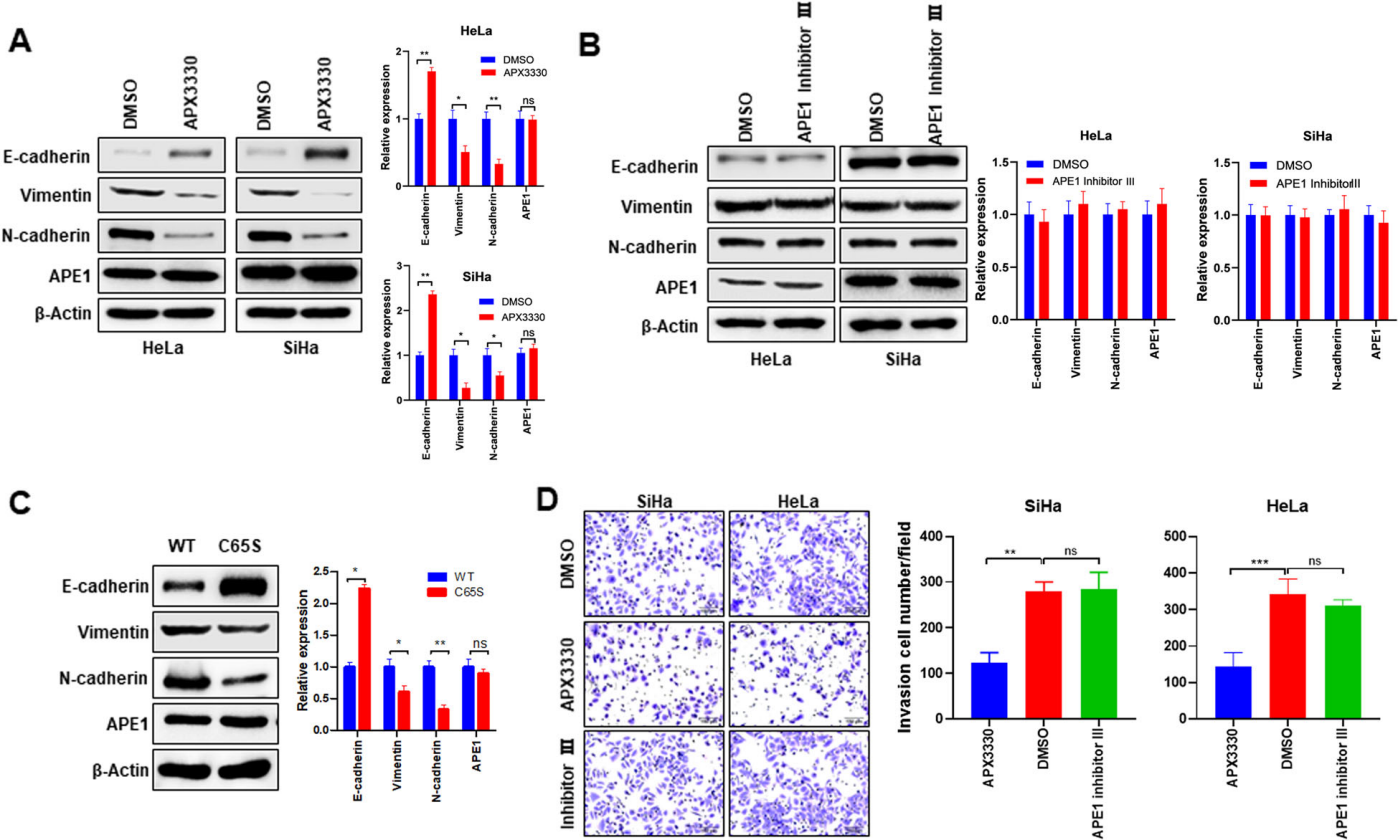
APE1 stimulates EMT and invasion through a redox-dependent mechanism in cervical cancer cells. **a** APX3330, an APE1 redox inhibitor, increased E-cadherin but inhibited N-cadherin and vimentin expression in cervical cancer cells. The indicated cells were treated with DMSO or 5 μM APEX3330 for 24 h and then subjected to western blot analysis. **b** Treatment of cervical cancer cells with APE1 inhibitor III did not affect EMT marker protein expression in cervical cancer cells. Cells were treated with DMSO or 7.5 μM APE1 inhibitor III for 24 h and then subjected to western blot analysis. **c** APE1 C65S, a mutant that ablates APE1 redox function, inhibited EMT compared to wild-type APE1. Western blotting was performed 72 h post-transfection. **d** Cervical cancer cell invasion was inhibited by APX3330 but not by APE1 inhibitor III. *, *P* < 0.05; **, *P* < 0.01; ***, *P* < 0.001

APE1 exerts functions both in the DNA repair response and redox regulation of transcription factors [19]. To investigate which function is involved in EMT regulation and invasion, cervical cancer cells were treated with the APE1 redox inhibitor (APX3330) [29] or the APE1 DNA repair inhibitor (APE1 inhibitor III) [30] and then subjected to EMT marker protein detection and invasion analysis. Our data show that APX3330 treatment increased E-cadherin expression but suppressed N-cadherin and vimentin expression in both HeLa and SiHa cells (Fig. 3a). In contrast, APE1 inhibitor III had no effect on EMT marker expression (Fig. 3b), despite impairing APE1 DNA repair activity (Fig. S3). Consistent with the APX3330 treatment, overexpression of mutant APE1, C65S, which lack redox function [31], increased E-cadherin expression and decreased N-cadherin and vimentin expression compared to overexpression of wild-type APE1 in HeLa cells (Fig. 3c). In addition, cell invasion assays also showed inhibition when challenged with APX3330 but not APE1 inhibitor III (Fig. 3d). Together, these findings suggest that APE1 stimulates cervical cancer cell EMT and invasion via a redox-dependent mechanism.

### APE1 inhibits E-cadherin by stimulating ZEB1 binding to the E-cadherin promoter

**Fig. 4:**
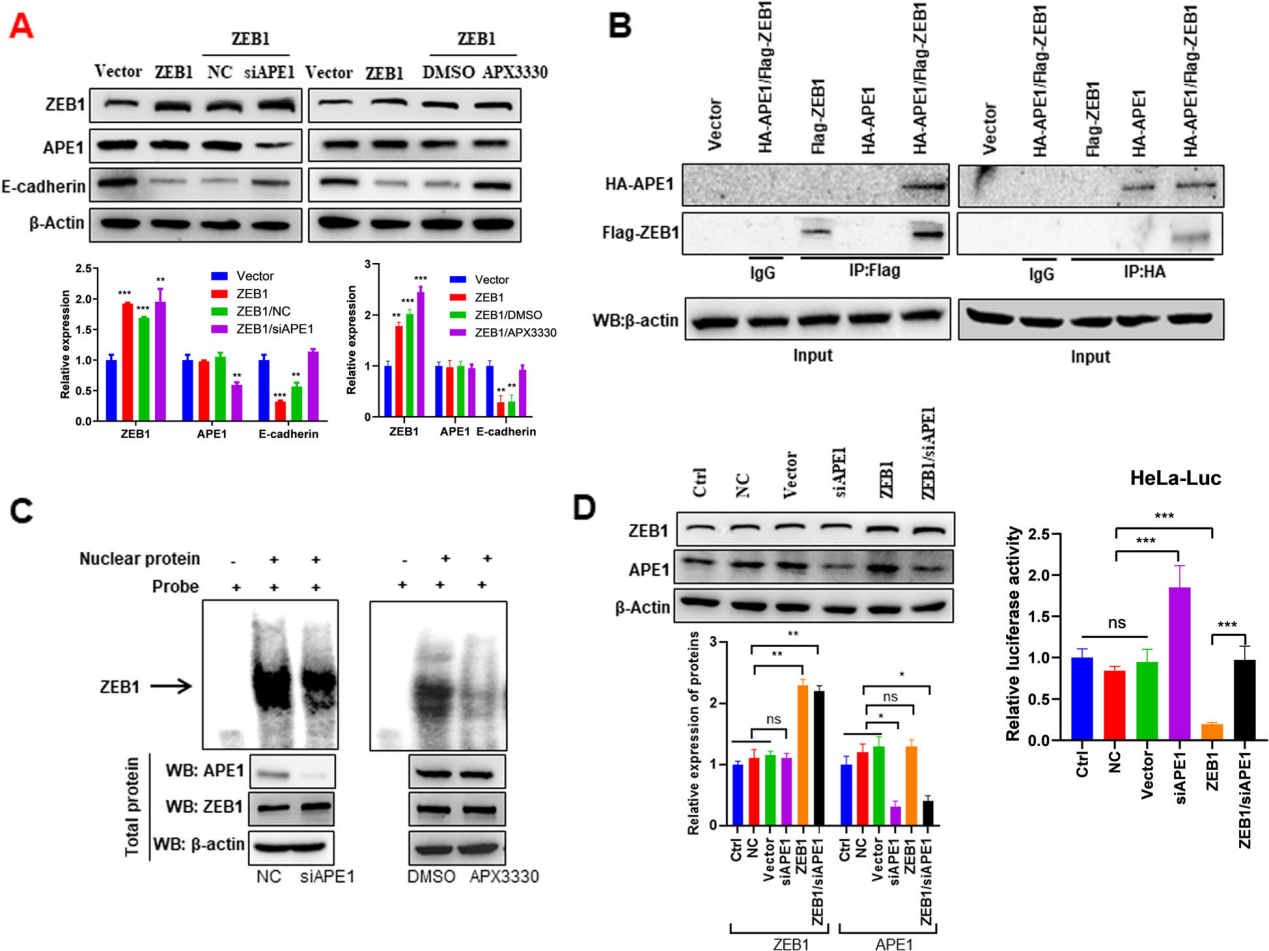
APE1 positively regulates ZEB1 and E-cadherin promoter binding in cervical cancer cells. **a** Silencing of APE1 or inhibition of APE1 redox function rescued ZEB1-mediated inhibition of E-cadherin expression in HeLa cells. Cells were transfected with indicated plasmid or nucleotides and Western blotting was performed after 72 h of transfection. For APE1 inhibitor experiment, cells were treated with 5 μM APX3330 or DMSO for 24 h after 48 h of transfection. **b** Co-IP showing that APE1 binds to ZEB1 in HeLa cells. HeLa cells were transfected with the indicated plasmids. After 72 h of transfection, cells were subjected to Co-IP. **c** EMSA showing that silencing APE1 or inhibition of the redox function of APE1 decreased ZEB1 and E-cadherin promoter binding in HeLa cells. HeLa cells were transfected with nontargeting siRNA or siRNA targeting APE1 for 72 h and then subjected to total protein or nuclear protein extraction; HeLa cells were treated with DMSO or 5 μM APX3330 for 24 h and then subjected to total protein or nuclear protein extraction. Western blots were conducted using total protein. **d** Silencing of APE1 rescued the ZEB1-mediated loss of luciferase activity in HeLa cells transfected with luciferase under the control of an E-cadherin promoter (HeLa-Luc). Western blot and luciferase assays were performed after 72 h of transfection. Vector, cells transfected with empty vector; NC, cells transfected with nontargeting control siRNA; Ctrl, cells not treated with anything. **, *P* < 0.01; ***, *P* < 0.001; ns, no significant difference

ZEB1 is an EMT-activating factor [32] that induces EMT through inhibiting E-cadherin expression by directly binding to the E-cadherin promoter [33]. Given that we showed that APE1 inhibits EMT by inhibiting E-cadherin expression in cervical cancer cells (Fig. 2b), we next investigated whether APE1 is involved in ZEB1-regulated inhibition of E-cadherin expression. The results showed that overexpression of ZEB1 inhibited E-cadherin expression and it was partially restored by silencing APE1 or APX3330 treatment in HeLa cells (Fig. 4a), indicating that APE1 was involved in ZEB1-regulated E-cadherin expression. In addition, Co-IP of Flag-ZEB1 or HA-APE1 expressed in HeLa cells showed a direct interaction between APE1 and ZEB1 (Fig. 4b). Importantly, EMSA results showed that APE1 silencing or APX330 treatment significantly reduced ZEB1 and E-cadherin promoter binding compared to controls (Fig. 4c), suggesting that APE1 positively regulates the interaction between ZEB1 and the E-cadherin promoter in a redox-dependent manner. As another means of confirmation, we constructed a reporter assay that relies on interactions between ZEB1 and the E-cadherin promoter to suppress the expression of luciferase in HeLa cells. Our luciferase reporter assay showed that ectopic expression of ZEB1 inhibited luciferase activity but was restored by APE1 silencing (Fig. 4d). Taken together, these findings indicate that APE1 inhibits E-cadherin expression by enhancing interactions between ZEB1 and the E-cadherin promoter by directly binding to ZEB1.

**Fig. 5:**
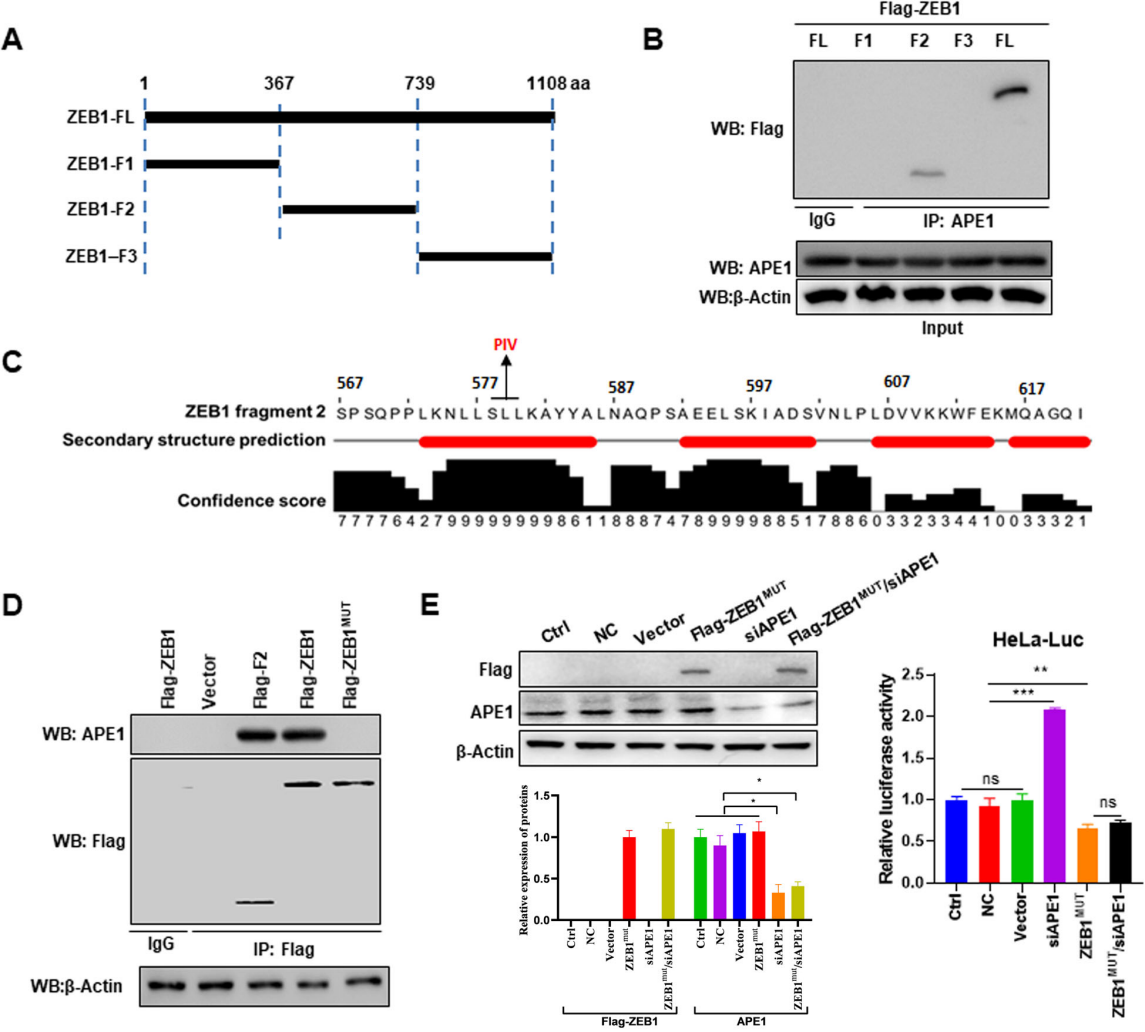
ZEB1 amino acids 578–580 are essential for the interaction with APE1. **a** Schematic representation of ZEB1 protein fragments. **b** Co-IP showing that the middle fragment of ZEB1 binds to APE1 (F2). Co-IP was performed after 72 h of transfection. **c** Secondary structure prediction of ZEB1-F2 suggesting the formation of an α helix. **d** Co-IP showing that mutation of ZEB1 578–580 disrupts the ZEB1-APE1 interaction. Co-IP was performed after 72 h of transfection. **e** Silencing of APE1 is unable to rescue ZEB1 induced inhibition of luciferase activity in the setting of the ZEB1 578–580 mutation. Western blot and luciferase assays were performed after 72 h of transfection. FL, full-length ZEB1; F1, ZEB1 fragment 1; F2, ZEB1 fragment 2; F3, ZEB1 fragment 3; Vector, cells transfected with empty vector; NC, cells transfected with nontargeting control siRNA; Ctrl, cells not treated with anything. Data are presented as the mean ± SD. ***, *P* < 0.001

Next, to map the interaction domain of ZEB1, truncated versions of ZEB1 (Fig. 5a) were expressed in HeLa cells, and Co-IP experiments evaluating ZEB1-APE1 interactions were performed. Among the fragments, only the ZEB1 fragment encompassing 368–739 amino acids was able to pull down APE1 (Fig. 5b). We noted that secondary structure prediction of that region of ZEB1 suggested that residues 573–621 had a high likelihood of forming an alpha helix (Fig. S4). We considered that the alpha helix might be important for binding to APE1 and designed mutations in residues 578–580 to disrupt the binding (Fig. 5c and Fig. S5). Co-IP showed that the mutated version of ZEB1, ZEB1^MUT^, was expressed but was unable to bind to APE1 (Fig. 5d). Additionally, we tested ZEB1^MUT^ in the E-cadherin promoter-driven luciferase reporter assay. As with wild type, ZEB1^MUT^ overexpression inhibited E-cadherin promoter-driven luciferase expression (Fig. 5e). However, this was not rescued by APE1 silencing (Fig. 5e), supporting that the helix formed by ZEB1 residues 573–621 is important for the interaction between ZEB1 with APE1 and resulting functional activity at the E-cadherin promoter.

### Inhibition of APE1 redox function suppressed lymph node and distant metastasis of cervical cancer cells in vivo

**Fig. 6:**
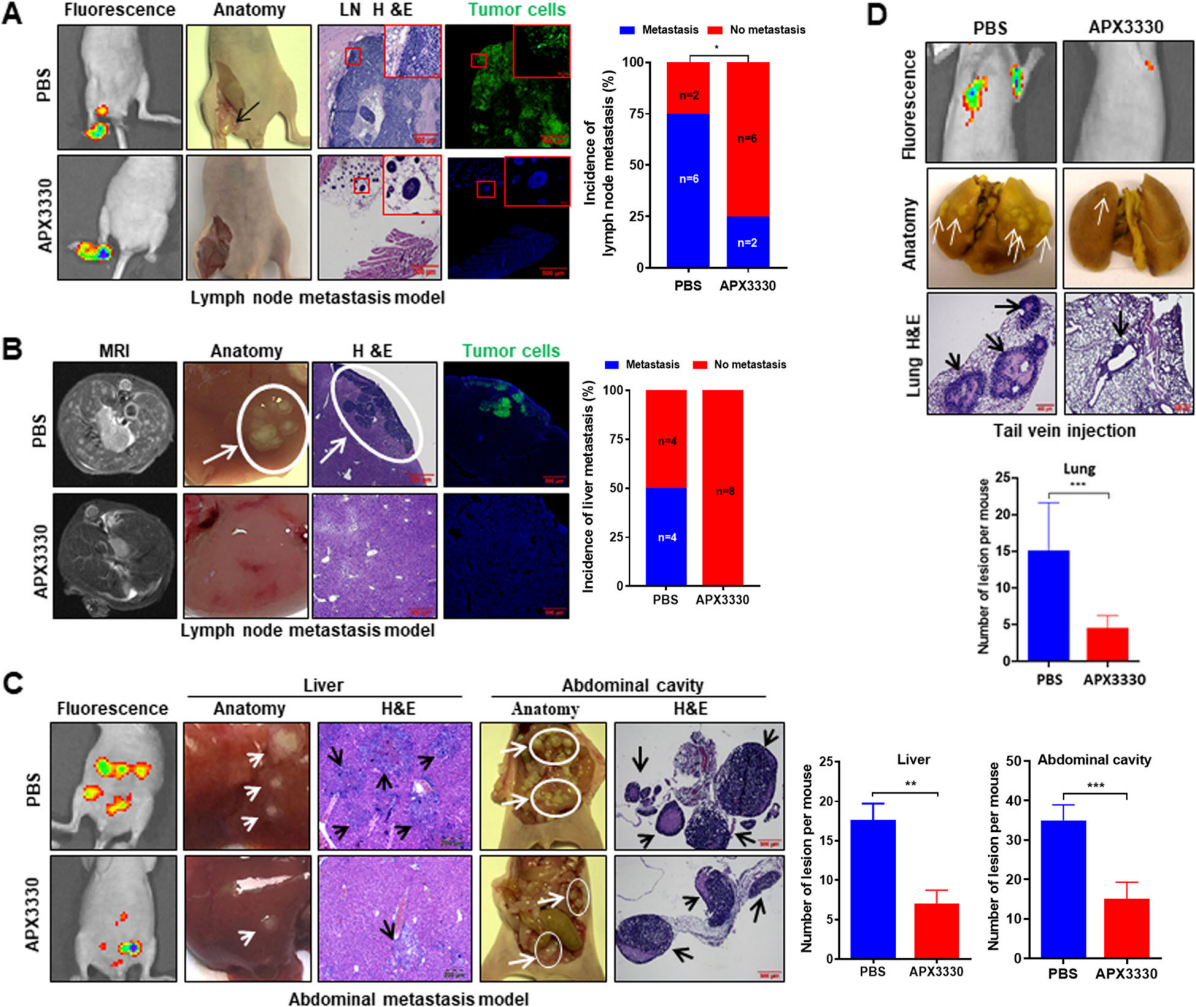
APX3330 suppressed cervical cancer metastasis in vivo. **a** APX3330 treatment reduced the incidence of lymph node metastasis in lymph node metastasis models. Lymph node metastatic models were generated with GFP-expressing HeLa cells (HeLa-GFP). Fluorescence image: Tumor metastasis was monitored by fluorescence imaging at day 28 after cell injection; Anatomic image: Arrow indicates metastatic tumors; LN H&E: Hematoxylin and eosin (H&E) staining of lymph nodes (LNs); Tumor cells: Green color indicates GFP-expressing HeLa cells in lymph nodes. Blue, DAPI. Data were tested by the chi square test. **b** APX3330 treatment inhibited the incidence of liver metastasis in cervical cancer lymph node metastasis models. MRI image: Liver metastasis of tumor was detected by MRI at day 28 after cell injection; Anatomic image: The circle and arrow indicates metastatic tumor nodules on the surface of the liver; H&E: H&E staining of liver tissues; The circle and arrow indicates metastatic lesions in the liver. Tumor cells: Green indicates GFP-expressing HeLa cells in the liver. Blue, DAPI. Data were tested by the chi square test. **c** APX3330 treatment reduced cervical cancer abdominal cavity metastasis. Xenograft models were generated by intraperitoneal injection of HeLa-GFP cells into mice. Fluorescence image: Tumor metastasis was monitored by fluorescence imaging on day 21 after cell injection. Anatomic image: Arrow indicates metastatic tumors. H&E: Hematoxylin and eosin (H&E) staining shows metastatic lesions in the abdominal cavity. The circle and arrows indicate metastatic tumors. Data are presented as the mean ± SD and analyzed by unpaired *t*-tests. *, *P* < 0.05; **, *P* < 0.01; ***, *P* < 0.001. **d** APX3330 treatment inhibited cervical cancer cell lung metastasis. Lung metastatic models were generated by tail vein injection of HeLa-GFP cells into mice. Fluorescence image: Tumor metastasis was monitored by fluorescence imaging on day 28 after cell injection. Anatomic image: Arrow indicates metastatic tumor nodules on the lung surface. H&E: Hematoxylin and eosin (H&E) staining shows metastatic lesions in the lung. Arrows indicate the metastatic tumors. PBS, mice were treated with PBS by I.P. injection. **, *P* < 0.01; ***, *P* < 0.001

Our in vitro findings suggested a therapeutic opportunity for tumors overexpressing APE1 in the form of APE1 redox inhibitors such as APX3330. We therefore investigated whether APX3330 could suppress cervical cancer cell metastasis in vivo. An established model for studying lymph node metastases in cervical cancer was generated by the injection of GFP-expressing HeLa cells into the footpad of nude mice [26]. Treatment of such mice with APX3330 showed a dramatic reduction in the incidence of nodal metastases at the 3-week mark, with 75% of the mice in the control group developing lymph node metastasis versus only 25% of the mice in the APX3330 treatment group (Fig. 6a). MRI and pathological examination showed that 50% of the mice in the control group developed hepatic metastasis, but no hepatic metastasis was found in the APX3330 treatment group (Fig. 6b). These results were further confirmed in models of systemic metastases, including a model of liver, colon and mesenteric metastases (Fig. 6c) and lung metastases (Fig. 6d). All in vivo experiments showed that APX3330 treatment did not affect the body weight of the experimental animals (Fig. S6). Taken together, these data suggest that inhibition of APE1 redox function by APX3330 may be a therapeutic strategy for the treatment of cervical cancer metastasis.

## Discussion

Lymph node and distant metastasis are major drivers of poor outcomes of cervical cancer [10, 34, 35]. Thus, a comprehensive understanding of the mechanisms underlying the development of metastasis is required to optimize treatment strategies and develop new therapeutic agents for cervical cancer. A previous study has shown that EMT is a primary process that involves increased cervical cancer metastasis with loss of epithelial markers, such as E-cadherin, and a gain of mesenchymal markers, such as vimentin [10]. Here, we used clinical sample analysis to indicate that high expression of APE1 is closely associated with lymph node metastasis and low expression of E-cadherin in cervical cancer patients (Fig. 1), and in vitro functional experiments indicated that overexpression of APE1 significantly stimulates cervical cancer cell invasion, promotes the vimentin expression, and inhibits E-cadherin expression, while silencing of APE1 dramatically inhibits cervical cancer cell invasion (Fig. 2). Together, these findings suggest that aberrantly increased expression of APE1 in cervical cancer promotes metastasis by stimulating EMT, and APE1 is a target for the treatment of cervical cancer metastasis.

Next, we elucidated the mechanism by which APE1 stimulates EMT in cervical cancer. Studies have shown that binding to ZEB1 and a dependence on ZEB1 to inhibit E-cadherin expression is a mechanism by which oncogenes promote EMT and metastasis in cancer. For example, telomerase reverse transcriptase (TERT) stimulates EMT in colorectal cancer through inhibition of E-cadherin expression by binding to ZEB1 [36]; histone H4K20-specific methyltransferase SET8 stimulates EMT in prostate cancer by inhibiting E-cadherin transcription through binding to ZEB1 [37]. Here, we found that APE1 also inhibits E-cadherin expression through a similar mechanism in cervical cancer. Our data clearly showed that APE1 stimulates ZEB1 and E-cadherin promoter binding by directly binding to ZEB1, thereby inhibiting E-cadherin expression in cervical cancer cells (Figs. 4 and 5). To our knowledge, this is the first evidence that APE1 stimulates EMT via direct binding to ZEB1.

Our findings may have therapeutic implications. One strategy might be to target ZEB1, given that ZEB1 is an essential driver of EMT activation and metastasis in cancer [38]. However, no specific ZEB1 inhibitors are available [38]. Here, we showed that targeting APE1 with the APE1 redox inhibitor APX3330 may be a feasible alternative. Our data showed that APX3330 inhibits ZEB1 and E-cadherin promoter binding, thereby restoring the E-cadherin expression inhibited by ZEB1 in cervical cancer cells (Fig. 4). Importantly, APX3330 treatment significantly inhibited cervical cancer cell EMT and invasion in vitro (Fig. 3) and cervical cancer lymph node and distant metastasis in vivo (Fig. 6). Our findings are supported by other research groups. Although the mechanisms are different, but the anti-EMT and anti-metastasis effects of APX3330 have been reported in various cancer types. For example, APX3330 treatment reverses the EMT phenotype in EGFR-mutated NSCLC via inhibition of TGF-β signaling [20]. Also, APX3330 treatment inhibits pancreatic cancer cell migration, but this was attributed to inhibition of STAT3 transcriptional activity [39]. Importantly, APX3330 is already in phase 1 clinical trials [40], suggesting the clinical potential of targeting APE1 by APX3330 in cervical cancer treatment, at least for APE high expressing patients.

## Conclusion

**Fig. 7:**
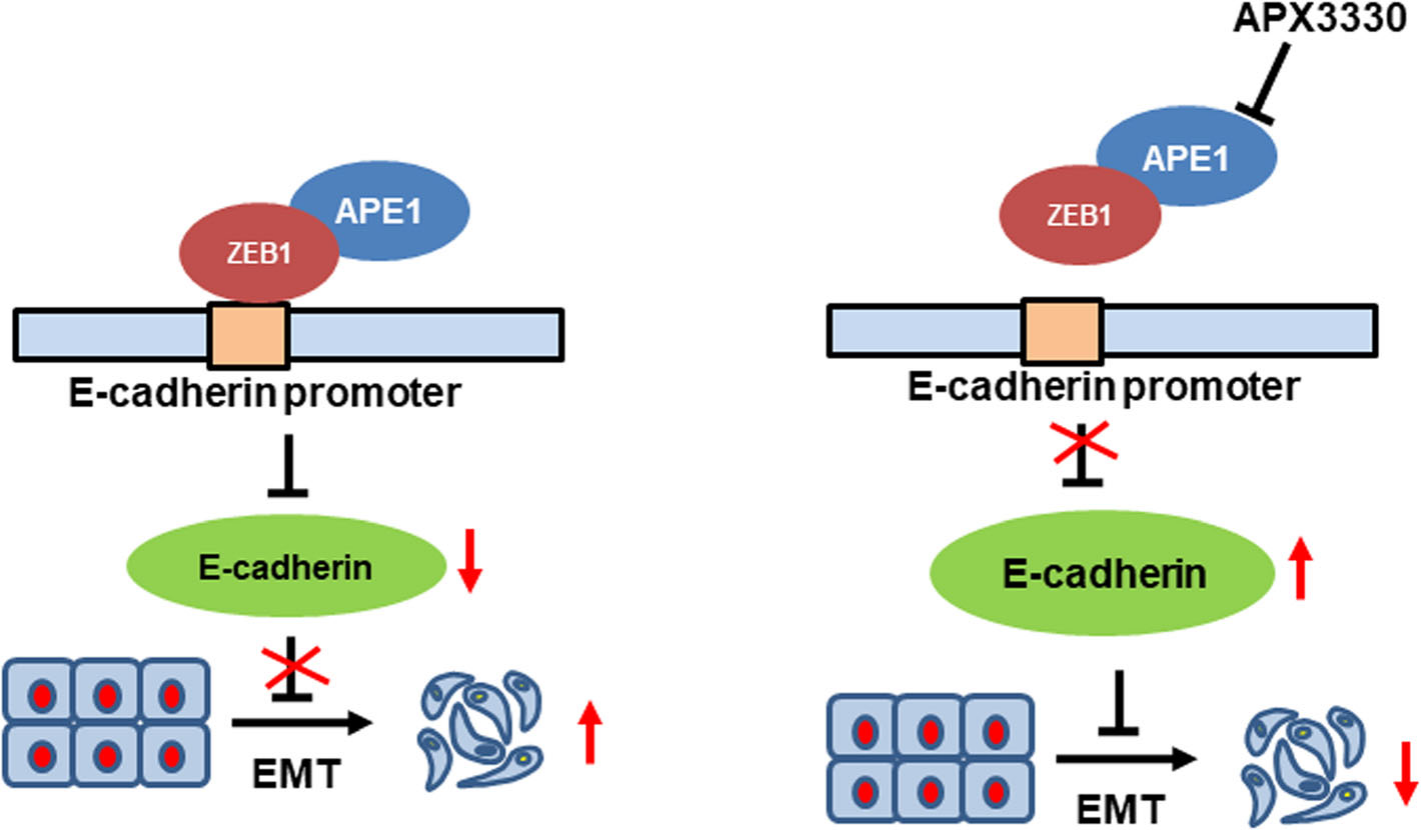
A schematic regulatory mechanism showing that inhibition of APE1 results in EMT suppression

Overexpression of APE1 is a promising biomarker for the management of cervical cancer because of its role in promoting cervical cancer metastasis. These findings establish a mechanistic link between APE1 and inhibition of E-cadherin expression; specifically, APE1 enhances the ZEB1 interaction with the E-cadherin promoter by directly binding to ZEB1 (Fig. 7). These findings also raise the possibility of new therapeutic opportunities in the form of APE1 inhibition.

## Supplementary Information

**Additional file 1: Supplementary Table 1.** Primer or probe sequences used in this study.

**Additional file 2: Fig. S1.** E-cadherin promoter sequence. **Fig. S2.** APE1 mRNA expression in cervical cancer tissues (Related to Fig. 1A). mRNA expression level of APE1 was measured by qRT-PCR and compared the expression level of APE1 between cancer tissues and adjacent tissues. If the APE1 expression level in cancer tissues was higher than adjacent tissues, it was classified as the APE1 high expression group and if there was no difference, it was classified as the APE1 low expression group. Data are presented as mean ± SD and analyzed by unpaired *t*-test. ***, *P* < 0.001. **Fig. S3.** APE1 inhibitor III dramatically inhibited DNA damage repair. HeLa and SiHa cells were transfected with APE1 expression vector. After 72 h of transfection, cells were treated with DMSO or 7.5 μM APE1 inhibitor III for 48 h then subject to DNA damage analysis. Data are presented as mean ± SD. **, *P* < 0.01; ***, *P* < 0.001. **Fig. S4.** Secondary structure prediction for second fragment of ZEB1. **Fig. S5.** Amino acids sequence of mutant ZEB1. **Fig. S6.** Body weight of experimental animals.

## Data Availability

All data generated of analyzed during this study are included in this published article and its supplementary information files. The datasets generated and used in this study are available from the corresponding author on reasonable request.

## Abbreviations

APE1: Apurinic/apyrimidinic endonuclease 1
ZEB1: Zinc finger E-box binding homeobox 1
EMT: Epithelial-Mesenchymal Transition
EMSA: Electrophoretic Mobility Shift Assay
