# Supplementary material for: Inhibiting the redox function of APE1 suppresses cervical cancer metastasis via disengagement of ZEB1 from E-cadherin in EMT
Source: J Exp Clin Cancer Res. 2021 Jul 1;40:220. doi: 10.1186/s13046-021-02006-5 (PMC8246661; doi:10.1186/s13046-021-02006-5)
Supplement: Supplementary file 1 — Additional file 1: Supplementary Table 1. Primer or probe sequences used in this study. [file 13046_2021_2006_MOESM1_ESM.docx]

**Supplementary Table 1. Primer or probe sequences used in this study**

| Gene | Primer sequences |
| --- | --- |
| ZEB1-F | Forward: 5’-GCAGGATCCATGAAAGTTACAAATTATAATACTGTG-3’  Reverse: 5’-GCAAAGCTTTTAGGCTTCATTTGTCTTTTCTTC-3’ |
| ZEB1-F1 | Forward: 5’-GCAGGATCCATGAAAGTTACAAATTATAATACTGTG-3’  Reverse: 5’-GCAAAGCTTACTGGTTGCCTGTAATGGGCCAC-3’ |
| ZEB1-2F | Forward: 5’-GCAGGATCCTCTCCTCAGGGCATGGTGCAAGCTGT-3’  Reverse: 5’-GCAAAGCTTAACACTAGTGATAGTTGACCTTTCTAA-3’ |
| ZEB1-3F | Forward: 5’-GCAGGATCCTACCAGAACAGTGTTTATTCTGTCCA-3’  Reverse: 5’-GCAAAGCTTTTAGGCTTCATTTGTCTTTTCTTC-3’ |
| E-cadherin promoter | Forward:5’- AAAGGGAGATCTGGCTGCTAGCTCAGTGGCTCATGG-3’  Reverse:5’-TTACACGGCGATCTTTCCGCCCTTC-3’ |
| EMSA probe 1 | Forward: 5’-TCCCATAACCCACCTAGACCCTAGC-3’  Reserve: 5’-GCTAGGGTCTAGGTGGGTTATGGGA-3’ |
| EMSA probe 2 | Forward: 5’-CTCCGGGGCTCACCTGGCTGCAGCCA-3’  Reserve: 5’-TGGCTGCAGCCAGGTGAGCCCCGGAG-3’ |

ZEB1-F, ZEB1 full length; ZEB1-F1, ZEB1 fragment 1; ZEB1-F2, ZEB1 fragment 2; ZEB1-F3, ZEB1 fragment 3

**Supplementary figure legends**

**Fig. S1** E-cadherin promoter sequence.

**Fig. S2** APE1 mRNA expression in cervical cancer tissues (Related to Fig. 1A). mRNA expression level of APE1 was measured by qRT-PCR and compared the expression level of APE1 between cancer tissues and adjacent tissues. If the APE1 expression level in cancer tissues was higher than adjacent tissues, it was classified as the APE1 high expression group and if there was no difference, it was classified as the APE1 low expression group. Data are presented as mean ± SD and analyzed by unpaired *t*-test. ***, *P*<0.001.

**Fig. S3** APE1 inhibitor III dramatically inhibited DNA damage repair. HeLa and SiHa cells were transfected with APE1 expression vector. After 72 hours of transfection, cells were treated with DMSO or 7.5 µM APE1 inhibitor III for 48 hours then subject to DNA damage analysis. Data are presented as mean ± SD. **, *P*<0.01; ***, *P*<0.001.

**Fig. S4** Secondary structure prediction for second fragment of ZEB1.

**Fig. S5** Amino acids sequence of mutant ZEB1.

**Fig. S6** Body weight of experimental animals.
