# Supplementary material for: Inhibiting the redox function of APE1 suppresses cervical cancer metastasis via disengagement of ZEB1 from E-cadherin in EMT
Source: J Exp Clin Cancer Res. 2021 Jul 1;40:220. doi: 10.1186/s13046-021-02006-5 (PMC8246661; doi:10.1186/s13046-021-02006-5)
Supplement: Supplementary file 2 — Additional file 2: Fig. S1. E-cadherin promoter sequence. Fig. S2. APE1 mRNA expression in cervical cancer tissues (Related to Fig. 1A). mRNA expression level of APE1 was measured by qRT-PCR and compared the expression level of APE1 between cancer tissues and adjacent tissues. If the APE1 expression level in cancer tissues was higher than adjacent tissues, it was classified as the APE1 high expression group and if there was no difference, it was classified as the APE1 low expression group. Data are presented as mean ± SD and analyzed by unpaired t-test. ***, P < 0.001. Fig. S3. APE1 inhibitor III dramatically inhibited DNA damage repair. HeLa and SiHa cells were transfected with APE1 expression vector. After 72 h of transfection, cells were treated with DMSO or 7.5 μM APE1 inhibitor III for 48 h then subject to DNA damage analysis. Data are presented as mean ± SD. **, P < 0.01; ***, P < 0.001. Fig. S4. Secondary structure prediction for second fragment of ZEB1. Fig. S5. Amino acids sequence of mutant ZEB1. Fig. S6. Body weight of experimental animals. [file 13046_2021_2006_MOESM2_ESM.pdf]

E-cadherin promoter region

-250 GGCGGGGCTG GGATTCGAACCCAGTGGAATCAGAACCGTGCAGGTCCCAT

-200 AACC CACCT AGACCCTAGCAACTCCAGGCTAGAGGGTCACCGCGTCTATG

-150 CGAGGCCGGGTGGGCGGGCCGTCAGCTCCGCCCTGGGGAGGGGTCCGCGC

-100 TGCTGATTGGCTGTGGCCGGCAGGTGAACCCTCAGCCAATCAGCGGTACG

-50 GGGGGCGGTGCCTCCGGGGCT CACCT GGCTGCAGCCACGCACCCCCTCTC

▭ : ZEB1 binding site; Red: EMSA probe

Fig. S1

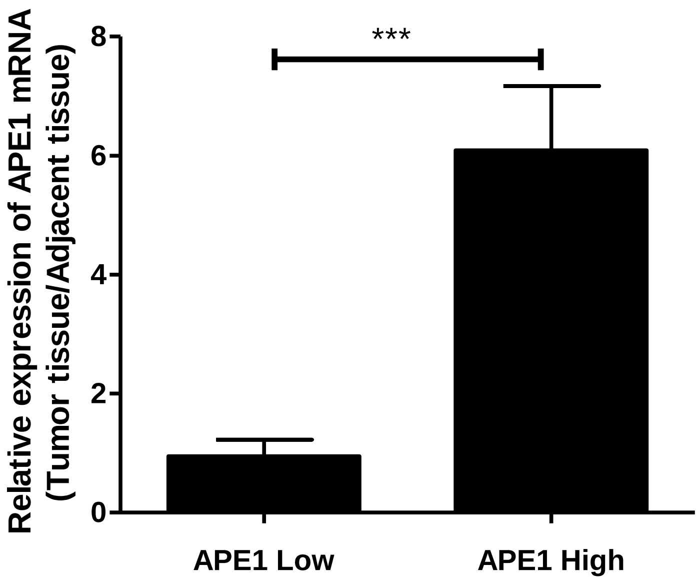
Relative expression of APE1 mRNA
(Tumor tissue/Adjacent tissue)
8
6
4
2
0
***
APE1 Low
APE1 High

Fig. S2

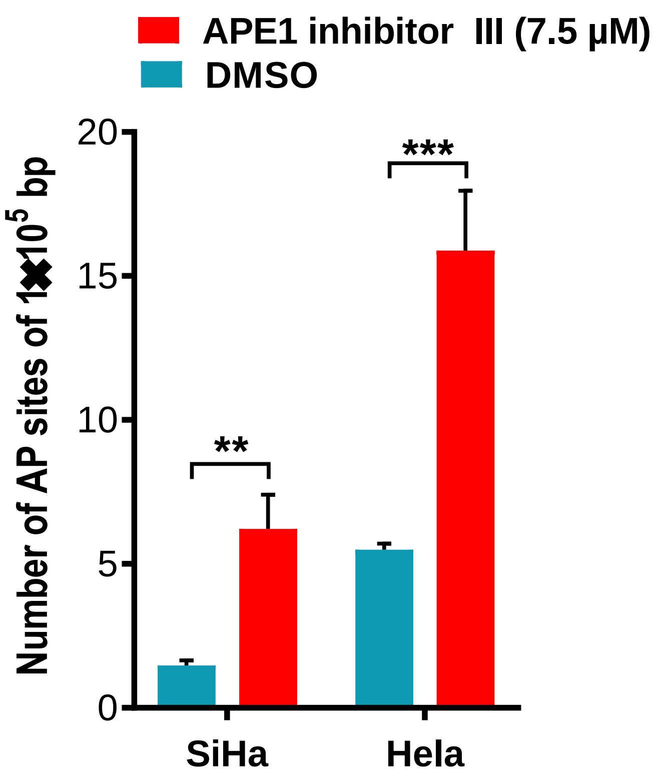
APE1 inhibitor III (7.5 μM)
DMSO
Number of AP sites of 1✖10⁵ bp
20
15
10
5
0
***
**
SiHa
Hela

Fig. S3

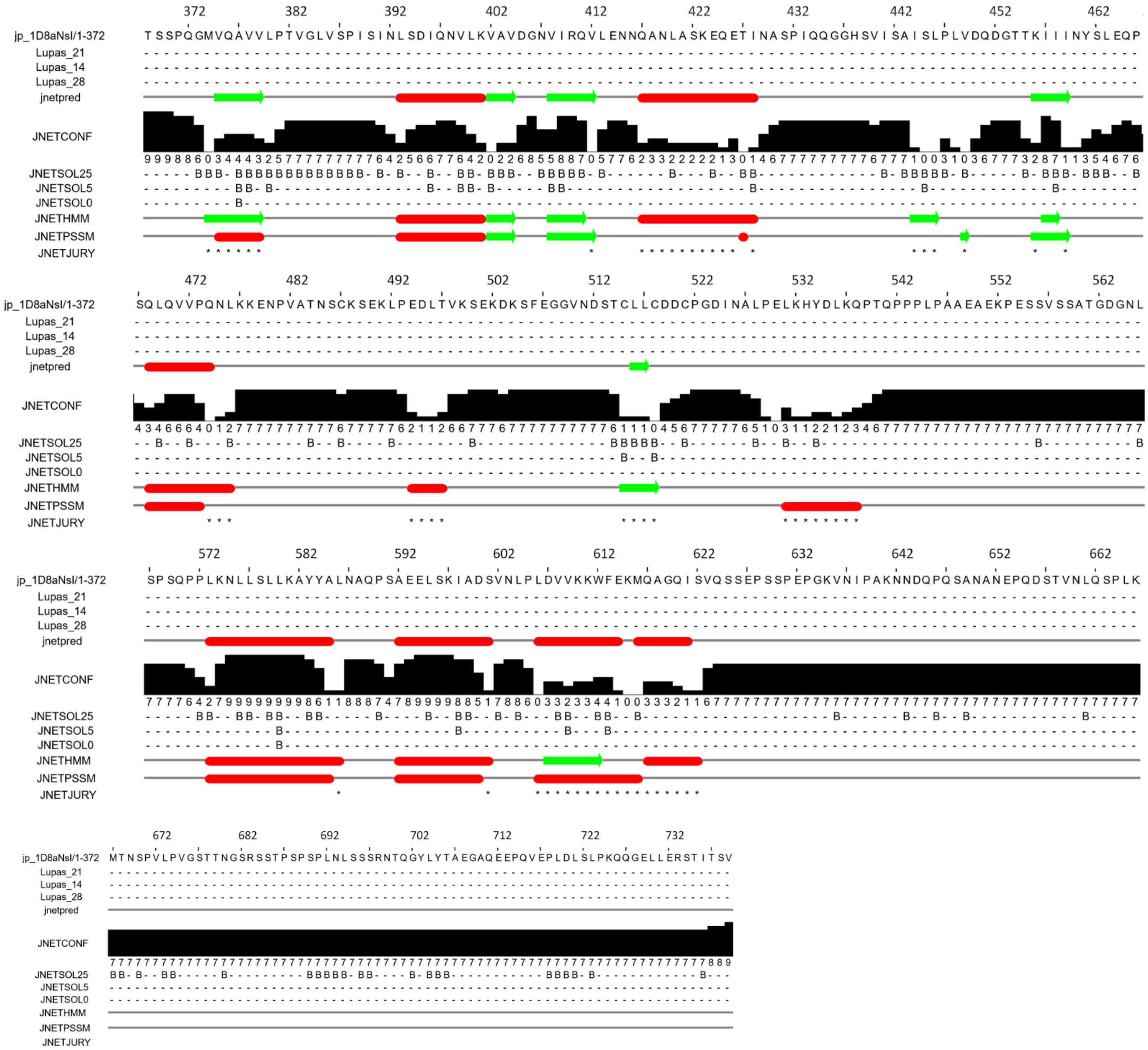

Fig. S4

561 TGDGNLSPSQ PPLKNLLSLL KAYYALNAQP Wild type

561 TGDGNLSPSQ PPLKNLLPIV KAYYALNAQP Mutant

Fig. S5

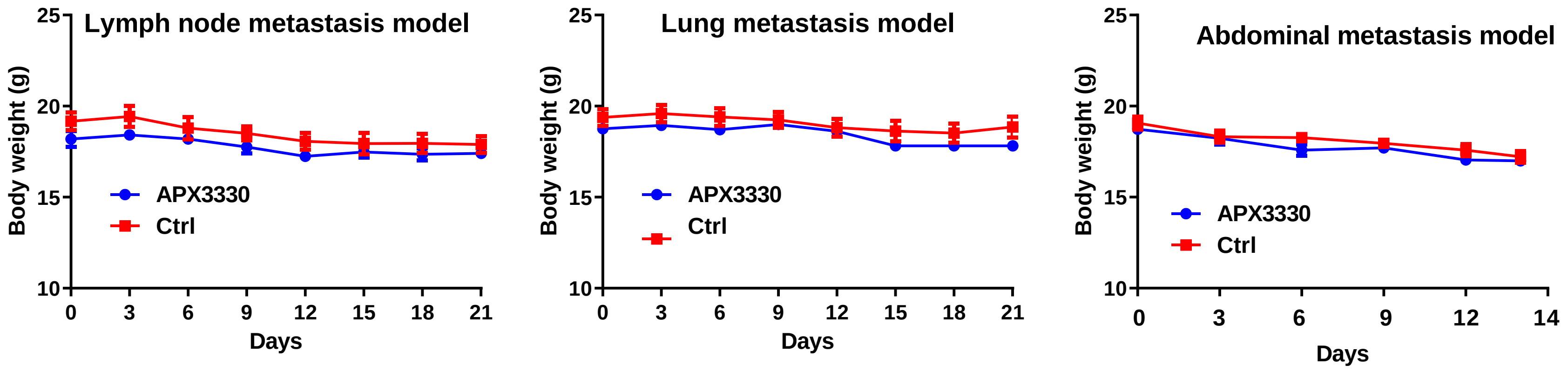

Lymph node metastasis model
Body weight (g)
25
20
15
10
0 3 6 9 12 15 18 21
Days
APX3330
Ctrl
Lung metastasis model
Body weight (g)
25
20
15
10
0 3 6 9 12 15 18 21
Days
APX3330
Ctrl
Abdominal metastasis model
Body weight (g)
25
20
15
10
0 3 6 9 12 14
Days
APX3330
Ctrl

Fig. S6
